# Sex difference in the risk for exercise-induced albuminuria correlates with hemoglobin A1C and abnormal exercise ECG test findings

**DOI:** 10.1186/s12933-017-0560-4

**Published:** 2017-06-23

**Authors:** Rafael Y. Brzezinski, Inbal Etz-Hadar, Ayelet Grupper, Michal Ehrenwald, Itzhak Shapira, David Zeltser, Shlomo Berliner, Ori Rogowski, Roy Eldor, Shani Shenhar-Tsarfaty

**Affiliations:** 1 Department of Internal Medicine “C”, “D” and “E”, Tel Aviv Sourasky Medical Center and Sackler Faculty of Medicine, Tel Aviv University, 6 Weizmann Street, 64239 Tel Aviv, Israel; 2 Nephrology Department, Tel-Aviv Sourasky Medical Center and Sackler Faculty of Medicine, Tel-Aviv University, Tel-Aviv, Israel; 3 Diabetes Unit, Institute of Endocrinology, Metabolism and Hypertension, Tel Aviv Sourasky Medical Center, Tel Aviv, Israel

**Keywords:** Albuminuria, Exercise, Stress test, Sex difference, Gender, HbA1c, Microvascular, Women-heart disease, Risk scores

## Abstract

**Background:**

Albuminuria is an established marker for endothelial dysfunction and cardiovascular risk in diabetes and prediabetes. Exercise induced albuminuria (EiA) appears earlier and may be a more sensitive biomarker for renal endothelial damage. We sought to examine the association between EiA, parameters of the metabolic syndrome, A1C levels, exercise ECG test results and sex related differences in a large cohort of healthy, pre-diabetic and diabetic subjects.

**Methods:**

A total of 3029 participants from the Tel-Aviv Medical Center Inflammation Survey cohort (mean age 46 years, 73% men) were analyzed. Multiple physiologic and metabolic parameters including A1C were collected and albuminuria was measured in all subjects before and immediately after completing an exercise ECG test.

**Results:**

Exercise increased urinary albumin to creatinine ratio (ΔEiA) by 2.8 (0–13.6) mg/g for median (IQR) compared to rest albuminuria (p < 0.001). An increase in ΔEiA was observed with accumulating parameters of the metabolic syndrome. ΔEiA showed significant interaction with sex and A1C levels; i.e. women with A1C > 6.5% had an increased risk of higher ΔEiA (p < 0.001). Using a cutoff of ΔEiA > 13 mg/g (top quartile) we found that women with ΔEiA > 13 mg/g were at greater risk for abnormal exercise ECG findings, (OR = 2.7, p = 0.001).

**Conclusion:**

Exercise promotes excessive urinary albumin excretion in dysmetabolic patients. In women, a significant correlation exists between ΔEiA and A1C levels. A cutoff of ΔEiA > 13 mg/g in women may be used to identify populations at risk for abnormal exercise ECG test findings and perhaps increased cardiovascular risk. Future studies will be needed to further validate the usefulness of ΔEiA as a biomarker for cardiovascular risk in women with and without diabetes.

## Background

Diabetic nephropathy (DN) is the leading cause of renal disease in adults [1] and is characterized by two distinct biomarkers; estimated glomerular filtration rate (eGFR) and urinary albumin excretion (often assessed as albumin to creatinine ratio (UACR) ≥30 mg/g in a urine spot test). These two biomarkers provide the basis for the kidney disease: improving global outcomes (KDIGO) chronic kidney disease (CKD) classification [2]. DN, is associated with a significant increase in mortality, and has been identified as a leading cause of excess risk of death in patients with diabetes [3]. Nevertheless and despite clear screening recommendations the number of patients with under diagnosed DN remains substantially high [4].

Several studies have shown that exercise unmasks albuminuria in normo-albuminuric diabetic individuals (exercise induced albuminuria—EiA, measured as spot UACR before and after exercise), suggesting this as the earliest sign of DN [5, 6]. Exercise leads to albuminuria by both modulating glomerular passage and tubular reuptake of albumin [7, 8]. We have recently published a study showing that EiA is independently correlated with a higher prevalence of metabolic syndrome in non-diabetic individuals, however the association among diabetic and pre-diabetic patients in a sex related perspective has never been studied [9].

Clear sex related differences have been identified in cardiovascular disease (CVD) [10, 11]. The clinical presentation of ischemic heart disease in women is very different from the classical presentation described in men and is therefore under diagnosed [12, 13]. This may result from pathophysiologic differences with a greater contribution of microvascular myocardial damage to disease progression in women [14, 15]. Recent studies have demonstrated sex differences in renal damage among healthy and diabetic individuals [16]. In non-diabetic individuals with chronic kidney disease, male sex has been identified as a risk factor for rapid decline in renal function as opposed to pre-menopausal women [17]. In diabetic individuals conflicting reports exist in different populations; a recent study among Japanese subjects with type 2 diabetes identified a higher risk in women [18], whereas a report regarding European individuals indicated an increased risk for renal decline in men [19]. Thus, the influence of sex on the development of DN has yet to be established conclusively [16, 20–23].

We, therefore, sought to investigate the association between EiA, sex, metabolic and physiologic parameters in a large cohort of individuals (both healthy, pre-diabetic and diabetic) who participated in the Tel-Aviv Medical Center Inflammation Survey.

## Methods

### Population

We analyzed data that was collected between January 2012 to September 2016 at the Tel-Aviv Medical Center Inflammation Survey, a registered databank of the Israeli Ministry of Justice [24–27]. The Tel-Aviv Medical Center Inflammation Survey encompasses a large cohort of subjects who attended the Tel-Aviv Medical Center for routine annual checkups (including a physician’s interview and examination, blood and urine tests, and an exercise stress test). The study was approved by the local Ethics committee and informed consent was obtained from all participants.

For the purpose of this study, urine samples for albumin and creatinine measurements were collected from participants before and after an exercise stress test was performed according to the Bruce protocol. A total of 3712 subjects were included in our database. We excluded 185 subjects due to lack of urinary tests (before or after the exercise test), and another 342 subjects without valid exercise tests results (test performed on a bicycle instead of a treadmill or an incomplete test). After these exclusions, the final cohort comprised 3185 participants. Another 156 individuals had no valid measurements of urinary creatinine concentration before or after the exercise, therefore, the values shown in Table 1 include only 3029 subjects.

**Table 1:** Population characteristics by sex

Characteristic	Men	Women	p value
Number of subjects	2218	811	
Age, years	46.97 ± 10.5	45.6 ± 10.3	0.001
BMI, kg/m^2^	26.4 ± 4.0	24.6 ± 4.4	<0.001
METs	12.3 ± 3.8	10.2 ± 4.6	<0.001
eGFR, ml/min/m^2^	82.7 ± 14.2	81.9 ± 16.2	0.178
HemA1c, %	5.4 ± 0.5	5.4 ± 0.4	0.014
Baseline U albumin, mg/l	2.9 (0.8 to 7.6)	3.4 (0.8 to 8.2)	0.136
Following exercise U albumin, mg/l	5.3 (1.4 to 17.8)	4.2 (1.1 to 10.5)	0.027
Change in U albumin mg/l	13.6 ± 54.3	6 ± 48.9	<0.001
Baseline UACR, mg/g	2.9 (1.3 to 6.8)	4.8 (1.9–11.2)	<0.001
Following exercise UACR, mg/g	7.1 (2.5 to 22.3)	7.9 (2.9–22.3)	0.306
Change in UACR, mg/g	3.1 (0.1 to 14.7)	1.7 (−0.8 to 10.7)	0.003
Fasting glucose, mg/dl	88.4 ± 14.5	84.9 ± 12.4	<0.001
Diabetes diagnosis %	5%	3.7%	0.183
Systolic BP, mmHg	127.4 ± 14.3	118.8 ± 15.7	<0.001
Diastolic BP, mmHg	79.1 ± 9.7	74.3 ± 9.3	<0.001
Basal heart rate, BPM	68.7 ± 11.8	73.1 ± 11.7	<0.001
Total cholesterol, mg/dl	184.3 ± 32.5	190.9 ± 33.1	<0.001
High-density lipoprotein-cholesterol, mg/dl	48.8 ± 11.3	64.1 ± 16.4	<0.001
Low-density lipoprotein-cholesterol, mg/dl	112.1 ± 28.2	107.9 ± 28.0	<0.001
Triglycerides, mg/dl	118.8 ± 71.8	94.3 ± 47.0	<0.001
Blood creatinine, mg/dl	1.1 ± 0.1	0.8 ± 0.1	<0.001

Values are presented as mean ± SD, or median (interquartile range) for irregular distributed parameters

*BMI* body mass index, *A1C* hemoglobin A1C, *BPM* beats per minute, *BP* blood pressure, *U* urinary, *METs* metabolic equivalents, *UACR* urinary albumin to creatinine ratio, *eGFR* estimated glomerular filtration rate

### Study procedures

Blood samples were drawn upon the participants’ arrival to the center after a 12-h overnight fast. Only after the morning blood test participants were allowed to drink, and after the exercise test they were invited to eat breakfast. Urine tests were analyzed by an ADVIA chemistry analyzer (Siemens Healthcare Diagnostics, Tarrytown, NY, USA), which is an improved albumin detection method with an extended analytic range of 3–420 mg/l and coefficient of variation of 2% [28]. It is accepted that a test for UACR is the standard method for assessing proteinuria using a spot examination, as advised by National Kidney Foundation Kidney Disease Outcomes Quality Initiative guidelines [29]. Therefore, we performed calculation of the UACR with a standard cutoff of 30 mg/g for moderate albuminuria. eGFR values were calculated using the CKD-EPI formula [30].

Separation of A1C from non-glycated hemoglobin of whole blood samples in EDTA was done by Tosoh’s G7 HPLC (Tosoh Bioscience, Inc. San Francisco, CA, USA). The concentrations of glycated hemoglobin (A1C) and the concentration of total hemoglobin were measured, and the ratio reported as % A1C. A1C levels were categorized into three states, healthy; <5.7%, pre-diabetic; 5.7–6.4% and diabetic; >6.5% according to the American Diabetes Association (ADA) guidelines [31].‬‬‬‬‬‬‬‬‬‬‬‬‬‬‬‬‬‬‬‬‬‬‬‬‬‬‬‬‬‬‬‬‬‬‬‬‬‬‬‬‬‬

Evaluation and diagnosis of metabolic syndrome (and it’s components) was performed based on the joint interim statement of the International Diabetes Federation Task Force on Epidemiology and Prevention; National Heart, Lung, and Blood Institute; American Heart Association; World Heart Federation; International Atherosclerosis Society; and International Association for the Study of Obesity [32]. Briefly, elevated waist circumference was defined as ≥94 cm (37 in.) in men and ≥80 (31.5 in.) in women, as recommended for individuals of European and Middle Eastern descent. Elevated triglycerides were defined as ≥150 mg/dl (1.7 mmol/l) or on drug treatment for elevated triglycerides. Reduced high-density lipoprotein-cholesterol (HDL) was defined as <40 mg/dl (1.0 mmol/l) in men and <50 mg/dl (1.3 mmol/l) in women. Elevated blood pressure was defined as ≥130 mmHg for systolic blood pressure or ≥85 mmHg for diastolic blood pressure or on antihypertensive drug treatment in a patient with a history of hypertension. Elevated fasting glucose was defined as ≥100 mg/dl (5.55 mmol/l). The diagnosis of metabolic syndrome was based on the existence of at least three abnormal findings out of the five mentioned above.

Exercise ECG stress test was preformed according to the Bruce protocol. ECG results were manually reviewed on the spot by a cardiologist. The remarks were filled into the patient’s file and were categorized into two groups. The first was labeled “negative exercise ECG findings”, stating that there were no significant findings in the patient’s test. The second group was titled “positive exercise ECG findings”; stating that the patient’s test demonstrated irregular findings possibly indicating myocardial ischemia or cardiac arrhythmia. These findings included ST segment depressions in variating lengths and position, T wave abnormality and incidence of ventricular premature contractions (VPCs).

All participants were asked if they had any previous diagnosis of diabetes or cardiac disease as part of their medical history questioner. 160 Patients (133 males and 27 females) with reported history of ischemic heart disease were excluded from the ECG stress test analysis.

### Statistical analysis

All continuous variables are displayed as means (SD) for normally distributed variables or median [interquartile range (IQR)] for variables with abnormal distribution., whereas categorical variables are displayed as numbers (%) of patients within each group. The different biomarkers in men and women were compared by a Student’s *t* test for normally distributed variables and by the Mann–Whitney *U*-test for non-normally distributed variables. To assess associations among categorical variables, we used a χ^2^-test. The values before and after the exercise test were compared using the Wilcoxon signed ranks test.

A one-way ANOVA test was used to evaluate the difference in ΔEiA according to number of metabolic syndrome components.

A general linear univariate model was used to evaluate the estimated effect of specified covariates: age, sex, body mass index, metabolic equivalents (METs) achieved during the exercise stress test, eGFR and A1C levels using Parameter’s estimates; B (with 95% confidence intervals) for ΔEiA. Multivariate binary logistic regression was used to evaluate the odds ratio of positive ECG findings among subjects according to the presence of ΔEiA > 13 (mg/g) in men and women. The model was further adjusted for the following specified covariates: age > 46, BMI > 30, METs score, waist circumference, HDL, BP, glucose and triglycerides. p values of <0.05 were considered statistically significant. The IBM SPSS Statistics 22.0 statistical package was used to perform all statistical analyses (IBM Corporation, Armonk, New York, USA).

## Results

3029 participants were included in the analysis. Participant characteristics are shown in Table 1. For the entire cohort, urinary albumin concentrations were elevated after exercise; the median urinary albumin concentration before and after exercise were 3 mg/l (IQR; 0.8–7.8) and 5 (IQR; 1.5–15.5) mg/l respectively (p < 0.001). The change in urinary albumin concentrations was 1.3 (−0.7 to 8.6) mg/l for median (IQR). UACR values before and after exercise were 3.3 (1.4–7.8) and 7.5 (2.6–22.3) mg/g for median (IQR) respectively (p < 0.001). The exercise induced change in UACR (ΔEiA) was 2.8 (0–13.6) mg/g for median (IQR). To rule out the possibility of an increase in EiA due to dehydration, urine specific gravity was analyzed before and after exercise. No significant difference was demonstrated between before and after exercise values (1.0076 and 1.004 respectively, p value = 0.184). Furthermore, no significant correlation with albumin concentrations or exercise induced change in albumin was noted (r = 0.024 and 0.022, p value >0.2 for both).

A very weak positive correlation between A1C and baseline UACR was found for the entire cohort (r = 0.083, p < 0.001). This correlation increased slightly when albumin concentration was measured following exercise (r = 0.107, p value <0.001).

**Fig. 1:**
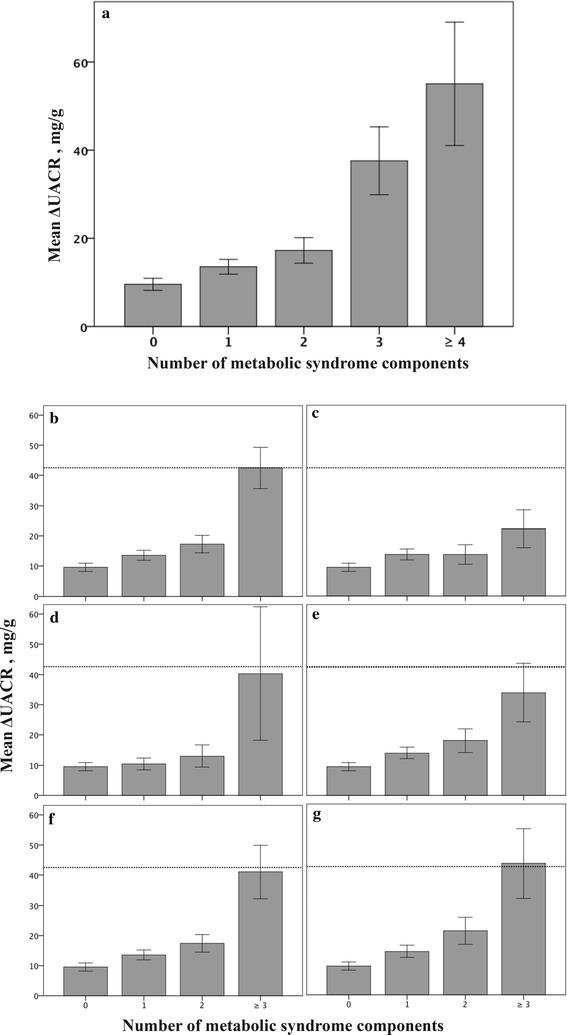
Change in urinary albumin to creatinine ratio following exercise (ΔEiA) according to number of metabolic syndrome components. **a** For the entire cohort, a significant increase of ΔEiA according to accumulating metabolic syndrome components was observed (p < 0.001). **b** For the entire cohort, reference line indicates mean ΔUACR = 42.5 SE ± 6.8 (mg/g) for metabolic syndrome patients (three components and higher). **c** After excluding patients with the *waist* criterion, lower levels were demonstrated, mean ΔUACR = 22.3, SE ± 6.2 (mg/g) for metabolic syndrome patients (p = 0.04). **d** Following exclusion of patients with the *blood pressure* criterion, **e** following exclusion of patients with the *triglycerides* criterion. **f** Following exclusion of patients with the *glucose* criterion, **g** following exclusion of patients with the *HDL* criterion. No substantial differences from the entire cohort were demonstrated after these exclusions (**d**–**g**)

A one-way ANOVA test demonstrated significant difference in ΔEiA with accumulating parameters of the metabolic syndrome; mean ΔEiA = 9.5 ± 1.4, 13.5 ± 1.7, 17.3 ± 2.9, 37.6 ± 7.7, 55.1 ± 14 (mg/g) (±SE) for patients with 0, 1, 2, 3, 4 or more metabolic syndrome components respectively, p < 0.001. This analysis was repeated, excluding patients with one of the four different metabolic syndrome components at a time. A similar pattern was demonstrated repeatedly with no significant differences in ΔEiA between the groups of exclusion. In the waist circumference criteria excluded group (651 patients excluded), a similar pattern was observed but lower values were shown; mean ΔEiA = 22.3 ± 6.2 (mg/g) for patients with the metabolic syndrome (three components or more) vs. 42.5 ± 6.8 (mg/g) for metabolic syndrome patients in the entire cohort (Fig. 1).

### Sex effect

Men were older, had higher BMI, METs score during the exercise test, blood pressure measurements, LDL, A1C and fasting glucose levels alongside lower levels of HDL (Table 1). At rest, men presented with lower UACR values compared to women; 2.9 (1.3–6.8) vs. 4.8 (1.9–11.2) mg/g for median (IOR), p < 0.001. Following exercise UACR values were similar; 7.1 (2.5–22.3) vs. 7.9 (2.9–22.3) mg/g for median (IQR), p = 0.306. The change in UACR values (ΔEiA) was significantly higher in men compared to women; 3.1 (0.1–14.7) vs. 1.7 (−0.8 to −10.7) mg/g for median (IQR).

General linear univariate model to predict baseline UACR, showed significant effect of eGFR, but no significant effect of sex, age, A1C levels, BMI or METs components (Table 2).

**Table 2:** Parameter estimates of general linear univariate model to predict rest albuminuria and ΔEiA

Dependent variable	Rest UACR			ΔEiA		
Predictors	B	CI (95%)	p value	B	CI (95%)	p value
Sex	32.95	−9.95 to 75.85	0.132	−89.98	−0.13 to −44.42	*<0.001*
Age	−0.1	−0.39 to 0.18	0.488	−0.1	−0.41 to 0.2	0.522
BMI	0.31	−0.3 to 0.92	0.319	2	1.34 to 2.66	*<0.001*
eGFR	−0.38	−0.57 to −0.19	*<0.001*	0.01	−0.2 to 0.21	0.956
A1C categorized	−15.69	−55.28 to 23.91	0.437	−109.82	−151.86 to −67.78	*<0.001*
METs	−0.48	−1.01 to 0.13	0.122	0.68	0.0–1.33	*0.04*
Baseline U albumin				−0.05	−0.09 to −0.01	*0.016*
Sex*A1c categorized	32.24	−75.74 to 10.92	0.143	95.37	49.34–141.39	*<0.001*

The model estimates predictors for rest urinary albumin to creatinine ratio (Rest UACR) and ΔEiA (UACR following exercise minus UACR at rest). Presented are results of a general linear regression model with Rest UACR (*left side*) or ΔEiA (*right side*) as the dependent variable and sex, age, BMI, eGFR, A1C, METs, rest UACR and the interaction between sex and categories of A1C as independent variables. Sex and the interaction between sex and A1C were significant predictors at the exercise induced model but not at rest

**Fig. 2:**
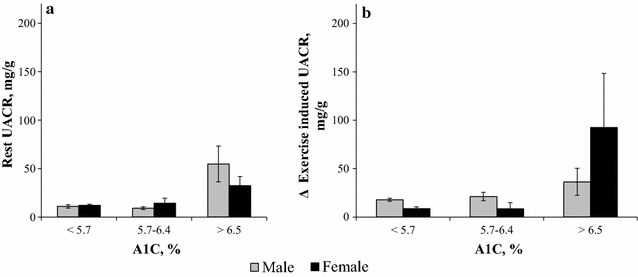
Rest and exercise induced albuminuria (EiA) according to categorized A1C levels and Sex. **a** Mean rest urinary albumin to creatinine ratio (UACR) according to A1C levels was similar between men and women, p = 0.143. **b** Following exercise women with A1C > 6.5% presented with a greater change in UACR (ΔEiA), p < 0.001

When searching for predictors of ΔEiA, we found a significant effect of sex, A1C and the interaction between them along with BMI, METs and baseline UACR (Table 2). Specifically, our data show that women with an A1C > 6.5% are at increased risk for higher ΔEiA even after controlling for age, BMI, eGFR, baseline UACR and METs; B = 95.37, 95% CI 49.34–141.39, p < 0.001 (Table 2; Fig. 2b).

**Fig. 3:**
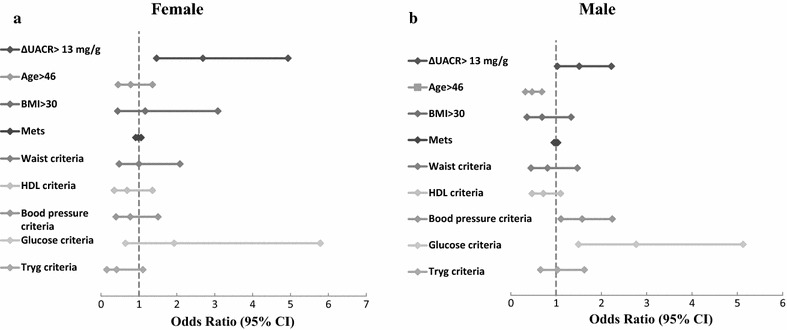
Multivariate predictors of positive exercise ECG findings. **a** Women with elevated change in urinary albumin to creatinine ratio following exercise (ΔEiA > 13 mg/g) demonstrated higher rates of positive exercise ECG findings (OR = 2.691, 95% CI 1.46–4.94, p = 0.00)1. **b** In men, a similar pattern was shown but with substantially reduced effect (OR = 1.511, 95% CI 1.02–2.22, p = 0.036). *Variables entered to the model were as following: ΔUACR > 13 mg/g, age > 46, BMI > 30, METs score, metabolic syndrome components; waist criteria, HDL criteria, BP criteria, glucose criteria and triglycerides criteria

A ΔEiA > 13 mg/g was defined as a cutoff level based on it being the lower bound of the top quartile of the cohort [75% of the cohort demonstrated ΔEiA < 13.6 (mg/g)]. ΔEiA > 13 mg/g was a significant predictor of positive (i.e. abnormal) exercise ECG findings in men and women, with women demonstrating much higher rates (OR = 2.691, p = 0.001 vs. OR = 1.511 p = 0.036, respectively). Among men, fasting glucose levels, Blood pressure and age were also observed as significant predictors (Fig. 3).

The positive predictive value (PPV) of ΔEiA > 13 mg/g in predicting positive ECG findings was higher in women compared to men (38 and 27% respectively). The negative predictive value (NPV) was high among the entire cohort NPV = 81% (82% in men and 77% in women).

## Discussion

CVD is the leading cause of morbidity and mortality among individuals with type 2 diabetes mellitus [33]. Among these patients, symptoms of coronary artery disease (CAD) are often absent even with advanced disease [34]. A wide variety of clinical strategies including exercise stress testing and various stress cardiac imaging techniques have been proposed for identifying diabetic patients with CAD before clinical manifestation is present [34–37]. In line with these, we suggest here ΔEiA as a sensitive and early biomarker for cardiovascular risk stratification in healthy, pre-diabetic and diabetic individuals.

Our data link ΔEiA to metabolic risk and clearly demonstrate a sex difference with regards to ΔEiA and the risk for an abnormal stress test. Women with diabetes are at a greater risk for higher ΔEiA which correlates with abnormal ECG findings during an exercise stress test. These differences were not detected by traditional biomarkers such as resting UACR and thus suggest a potential for ΔEiA as a biomarker for assessing CVD risk in women. Recent studies have shown similar patterns regarding exercise induced albuminuria and its correlation to the metabolic syndrome and diabetes in particular [5, 6, 9]. Our current study marks another step towards achieving a comprehensive understanding of dysmetabolic complications, their onset and progression throughout the disease. ΔEiA increased with accumulating metabolic syndrome criteria even after eliminating the effect of each criterion separately (with the largest effect observed after eliminating waist circumference criterion). Consequently, an increase in ΔEiA seems to be a characteristic disorder during progression of the metabolic syndrome.

Recent studies have shown that elevated low grade albuminuria (<30 mg/g) in the currently defined “normal” range is a predictor of worse CV outcomes [38–40]. These findings coupled with the currently reported findings linking high ΔEiA to increased CVD risk even in subjects with apparently normal albuminuria, call for a reassessment of our current definitions of “normal” range urinary albumin excretion.

The sex specific association between ΔEiA and an abnormal stress test ECG aligns with our current understanding of the role of microvascular heart disease in women with CVD. The observed link between these seemingly distinct processes is not surprising; measuring the effect of exercise on albuminuria is a sensitive method of directly assessing the renal microvasculature’s ability to withstand a “hemodynamic stress test”. It is reasonable to hypothesize that the metabolic process causing microvascular dysfunction would not be limited to the renal vasculature but affect other sensitive vascular beds including the heart. This concept has been recently demonstrated in other organs with microvascular disease such as retina and skin [41].

The major limitation of our study is the relative low event rate of major cardiovascular outcomes and evident renal damage in our cohort, limiting our assessment of the importance of ΔEiA in CV and renal disease risk stratification. Moreover, the current study was not designed to investigate whether ΔEiA predicts future CVD events or renal damage. Future longer studies with sufficient follow up periods, looking at major cardiovascular events (CV death, myocardial infarction and stroke) alongside incident albuminuria or reduced GFR, are needed to fully appreciate the potential of ΔEiA as a method for stratifying current and future CVD and CKD risk, and its clinical usefulness over widely used biomarkers such as resting UACR in women. However, the current findings in a relatively young and apparently healthy cohort were not detected by standard UACR and emphasize the ability to use ΔEiA as an early screening tool.

An additional limitation of our study is the relative wide definition of positive exercise ECG test findings; including ischemia related findings (ST segment depressions and T wave abnormalities) alongside VPCs. Future studies regarding EiA and CVD should address the debatable inclusion of VPCs under the definition of a positive cardiac stress test [42].

Another limitation of our study is the fact that a majority of women with A1C > 6.5% were post-menopausal. It is possible that sex hormones contribute to the progression of diabetic complications, specifically microvascular related ones [43]. There are numerous studies supporting this concept suggesting an influence of sex hormones on the renin-angiotensin system, endothelial function, and NO production (in relation to oxidative stress level) [44, 45]. A more focused investigation is needed to fully assess the contribution of sex hormones to ΔEiA and CVD.

A reduction in albuminuria is related to favorable cardiovascular outcomes [39, 46]. Early treatment of diabetic or hypertensive patients with angiotensin-converting enzyme inhibitors, angiotensin receptor blockers, β-blockers, Ca^2+^ channel blockers, DPP-4 inhibitors, GLP-1 agonists and more recently SGLT-2 inhibitors was effective in lowering rest albuminuria [47–51]. Our data suggests that stress test-induced albuminuria may serve as a powerful screening tool to detect a high-risk subpopulation of women who require careful follow-up and possible refinement of preventive treatment. On the contrary, the relatively high negative predictive value demonstrated in our findings suggest that evaluating ΔEiA could also serve as an important screening tool through which unnecessary and costly ECG exercise tests could be avoided in women.

## Conclusions

Our findings demonstrate excessive urinary albumin excretion following exercise among dysmetabolic patients. In women, ΔEiA is correlated with A1C levels. ΔEiA could possibly serve as a new screening tool for identifying female population at risk for abnormal exercise ECG test findings. The usefulness of ΔEiA as a biomarker for cardiovascular risk stratification among healthy and diabetic individuals will need to be validated in future studies.

## Abbreviations

CVD: cardiovascular disease
CAD: coronary artery disease
EiA: exercise induced albuminuria
A1C: glycated hemoglobin
UACR: urinary albumin to creatinine ratio
ΔEiA: UACR following exercise minus UACR at rest
DN: diabetic nephropathy
eGFR: estimated glomerular filtration rate
KDIGO: kidney disease: improving global outcomes chronic kidney disease classification
CKD: chronic kidney disease
ADA: American Diabetes Association
HDL: high-density lipoprotein-cholesterol
METs: metabolic equivalents score
VPC: ventricular premature contractions
SD: standard deviation
ANOVA: analysis of variance
CI: confidence Interval
OR: odds ratio
BMI: body mass index
IQR: inter quartile range
PPV: positive predictive value
NPV: negative predictive value
CVD: cardiovascular disease
